# The Role of Cadaverine Synthesis on Pneumococcal Capsule and Protein Expression

**DOI:** 10.3390/medsci6010008

**Published:** 2018-01-19

**Authors:** Mary F. Nakamya, Moses B. Ayoola, Seongbin Park, Leslie A. Shack, Edwin Swiatlo, Bindu Nanduri

**Affiliations:** 1 Department of Basic Sciences, College of Veterinary Medicine, P.O. Box 6100, Mississippi State, MS 39762, USA; mfn35@msstate.edu (M.F.N.); mba185@msstate.edu (M.B.A.); sp1679@msstate.edu (S.P.); shack@cvm.msstate.edu (L.A.S.); 2 Section of Infectious Diseases, Southeast Louisiana Veterans Health Care System, New Orleans, LA 70112, USA; edwin.swiatlo@va.gov; 3 Institute for Genomics, Biocomputing & Biotechnology, Mississippi State University, Mississippi State, MS 39762, USA

**Keywords:** *Streptococcus pneumoniae*, polyamines, pneumococcal pneumonia, proteomics, capsule, complementation, metabolism, cadaverine

## Abstract

Invasive infections caused by *Streptococcus pneumoniae*, a commensal in the nasopharynx, pose significant risk to human health. Limited serotype coverage by the available polysaccharide-based conjugate vaccines coupled with increasing incidence of antibiotic resistance complicates therapeutic strategies. Bacterial physiology and metabolism that allows pathogens to adapt to the host are a promising avenue for the discovery of novel therapeutics. Intracellular polyamine concentrations are tightly regulated by biosynthesis, transport and degradation. We previously reported that deletion of *cadA,* a gene that encodes for lysine decarboxylase, an enzyme that catalyzes cadaverine synthesis results in an attenuated phenotype. Here, we report the impact of *cadA* deletion on pneumococcal capsule and protein expression. Our data show that genes for polyamine biosynthesis and transport are downregulated in *∆cadA*. Immunoblot assays show reduced capsule in *∆cadA.* Reduced capsule synthesis could be due to reduced transcription and availability of precursors for synthesis. The capsule is the predominant virulence factor in pneumococci and is critical for evading opsonophagocytosis and its loss in *∆cadA* could explain the reported attenuation in vivo. Results from this study show that capsule synthesis in pneumococci is regulated by polyamine metabolism, which can be targeted for developing novel therapies.

## 1. Introduction

*Streptococcus pneumoniae* (pneumococcus) is a Gram-positive encapsulated pathogen that resides asymptomatically in the nasopharynx of healthy humans. In children, elderly, and immunocompromised individuals pneumococci can become pathogenic, causing mild to severe infections such as sinusitis, meningitis, community acquired pneumonia and septicemia [1]. Pneumococcal infections cause approximately 2 million deaths globally with about 900,000 reported cases in the United States alone, resulting in approximately 400,000 hospitalizations annually [2]. There are over 90 pneumococcal serotypes with unique capsular polysaccharide structures [3] and only 24 serotypes are included in the current existing polysaccharide-based vaccines, PCV13 and PPSV23 [4], combined. Increased resistance to antibiotics such as penicillin, cephalosporins, and fluoroquinolones also complicates treatment [5,6]. The diversity of pneumococcal serotypes, coupled with genomic plasticity and the increasing selection for non-vaccine serotypes, mandates the development of novel protein-based vaccines that are conserved across serotypes and drives the search for new antimicrobial targets.

Polyamines are small, ubiquitous polycationic molecules with hydrocarbon backbones that are positively charged at physiological pH. Polyamines participate in many important biological functions in both eukaryotes and prokaryotes [7]. They interact with negatively charged molecules such as nucleic acids, proteins, and modulate DNA replication, transcription and translation [8]. The most common cellular polyamines in prokaryotes are diamines, putrescine (1,4-diaminobutane) and cadaverine (1,5-diaminopentane), and a triamine, spermidine (*N*-(3-Aminopropyl)- 1,4-diaminobutane) [9,10,11]. Bacteria regulate intracellular polyamine levels by biosynthesis, import of extracellular polyamines through adenosine triphosphate (ATP)-binding cassette transporters (ABC transporters) or antiporters and catabolism.

Polyamines are known to modulate virulence of pathogenic bacteria [12]. Spermine influences biofilm formation in *Neisseria gonorrhoeae* [13], *Bacillus subtilis* [14], *Escherichia coli* [15], *Yersinia pestis* [16] and *Vibrio cholerae* [17]. Polyamines have been linked to escape from phagolysosomes, bacteriocin production, toxin activity and stress responses in many pathogenic bacteria [18,19,20,21]. Cadaverine inhibits multiplication of *Shigella flexneri* by preventing lysis of phagolysosome [20]. Cadaverine has also been shown to inhibit adherence of toxin producing *E. coli* [22]. Different types of polyamines have different effects on growth and virulence. Therefore, studies that focus on the impact of polyamines in bacterial pathogens and their role in infection are warranted. Polyamine transport and synthesis genes are highly conserved across pneumococcal serotypes [23], while polyamines catabolism is poorly annotated in the genome. Our earlier studies show that intact polyamine transport and synthesis genes are necessary for virulence of *S. pneumoniae* in murine models of colonization, pneumococcal pneumonia and sepsis [24]. Deletion of genes that encode lysine decarboxylase (*cadA*), and spermidine synthase (*speE*), enzymes that catalyze cadaverine and spermidine synthesis, and polyamine transporter (*potABCD*) in pneumococci results in an attenuated phenotype in vivo [24]. We also demonstrated the therapeutic potential of targeting polyamine metabolism genes in pneumococci. Immunization with PotD, the extracellular substrate binding subunit of the polyamine transport operon *potABCD*, affords protection in mice against colonization, pneumonia and sepsis [25,26]. However, the impact of the deletion of polyamine biosynthesis on pneumococcal protein expression, including virulence factor expression that ultimately regulates survival in the host in a polyamine dependent manner is not known [18].

Here, we investigated the role of *cadA*, on pneumococcal capsule and protein expression. We hypothesized that the attenuated phenotype of lysine decarboxylase deficient pneumococci is due to reduced capsule production, rendering the bacterium more susceptible to host defense. Our results show loss of thecapsule and downregulation of synthesis of putrescine and spermidine as well as transport in *ΔcadA*. Our data strongly suggests that polyamine metabolism plays a significant role in the regulation of capsular polysaccharide biosynthesis in pneumococci, and is an attractive target for developing novel therapeutics.

## 2. Materials and Methods

### 2.1. Bacterial Strains, and Growth Conditions

*Streptococcus pneumoniae* serotype 4 strain TIGR4 was used in this study [27]. All strains were grown in Todd-Hewitt broth supplemented with 0.5% yeast extract (THY) or on 5% sheep blood agar plates (BAP) in 5% CO_2_. An isogenic mutant of TIGR4 deficient in *cadA* was generated by polymerase chain reaction (PCR)-ligation mutagenesis as described previously [24]. Briefly, PCR primers were designed to amplify upstream (600 nt 5′ to the start codon) and downstream (600 nt 3′ from the transcription termination site) of *cadA* from TIGR4 chromosomal DNA (Table 1). Genomic pieces were joined by gene splicing, and insertion of spectinomycin resistance gene (*spec*) amplified from pORI280 [28] by overlap extension (SOEing) PCR [24]. The recombinant product was transformed into TIGR4 as described previously [29]. Transformants were selected on BAP with spectinomycin (100 μg/mL) and *cadA* gene deletion was confirmed by sequencing. To establish that cadaverine is responsible for loss of the capsule, we complemented the *ΔcadA* mutant in trans by cloning *cadA* gene amplified from TIGR4 (Table 1) into pABG5 vector for analysis of transcription [30], and complemented transformants were selected on BAP with kanamycin (50 μg/mL) and confirmed by PCR.

### 2.2. In Vitro Growth of TIGR4 and ΔcadA

TIGR4 and *ΔcadA* were inoculated into THY (10^5^ colony forming units (CFU)/mL) and growth was monitored by measuring optical density at 600 nm (OD_600 nm_) using a Cytation 5 multifunction plate reader (BioTek, Winooski, VT, USA) at 37 °C with 5% CO_2_. We used GrowthRates [31], a software tool that uses the output of plate reader files to automatically monitor growth rate in exponential phase, lag phase and maximal OD to compare TIGR4 and *ΔcadA* growth curves. We also measured the viability of TIGR4 and *ΔcadA* in THY by plating cells on BAP every 2 h for CFU enumeration. Morphology was compared by Gram staining of mid-log phase (OD_600 nm_ 0.4) cultures of bacteria.

### 2.3. Measurement of Capsular Polysaccharides

Capsular polysaccharide (CPS) was quantified by immunoblotting, as described [32]. An isogenic capsular variant of TIGR4 (T4R) in which the *cps* locus is replaced with the Janus cassette resulting in an unencapsulated phenotype [33] was used as a control. Briefly, bacterial strains were cultured in THY supplemented with 10% fetal bovine serum to an OD_600 nm_ of 0.2, plated on BAP for CFU enumeration and 1 mL bacteria was pelleted and stored at −20 °C until further use. The CFUs for all strains were ~ 9.0 × 10^7^/mL. CPS was extracted in a lysis buffer (4% deoxycholate, 50 μg/mL DNAseI and 50 μg/mL RNAse) at 37 °C for 10 min and centrifuged at 18,000× *g* for 10 min. Four threefold serial dilutions of the supernatant in phosphate-buffered saline (PBS) were spotted in duplicate (2 μL) on 0.2-μm-pore-size nitrocellulose membrane (Thermo Fisher Scientific, Waltham, MA, USA) with suction and air dried at 60 °C for 15 min. The membranes were blocked and incubated with rabbit anti-serotype 4 serum (Cedarlane, Burlington, NC, USA) at 1:1000 and a horseradish peroxidase (HRP) conjugated goat anti-rabbit secondary antibody (Thermo Fisher Scientific, Waltham, MA, USA) at 1:10,000. Membranes were developed with enhanced chemiluminiscence (ECL) detection (Thermo Fisher Scientific, Waltham, MA, USA) and scanned using a ChemiDoc XRS+ with Image Lab software (Bio-Rad, Hercules, CA, USA).

### 2.4. Proteomics

Total proteins were isolated from mid-log phase (OD_600 nm_ 0.4) TIGR4, and *ΔcadA* (*n* = 4) cultured in THY and subjected to liquid chromatography–tandem mass spectrometry (LC-MS/MS) analysis as described earlier [24,34]. Briefly, proteins were isolated from bacterial pellets sonicated in NP-40 lysis buffer (0.5% NP-40, 150 mM NaCl, 20 mM CaCl_2_·2H_2_O, 50 mM Tris, pH 7.4) supplemented with 1X protease inhibitor cocktail/ethylenediaminetetraacetic acid (EDTA) using a Covaris S220 focused-ultrasonicator (Covaris, Woburn, MA, USA). Protein concentration from the supernatant was determined using a Pierce bicinchoninic acid assay (BCA) Protein Assay Kit (Thermo Fisher Scientific, Waltham, MA, USA) and 30 µg was precipitated with methanol and chloroform (4:1), solubilized in 8 M urea, reduced (0.005 M dithiothreitol (DTT) at 65 °C for 10 min) and alkylated (0.01 M iodoacetamide at 37 °C for 30 min) and digested with porcine trypsin (2 µg at 37 °C, overnight, 50:1 ratio of protein: trypsin, Promega Corporation, Madison, WI, USA). Tryptic peptides were desalted using a C18 spin column (Thermo Fisher Scientific, Waltham, MA, USA) and analyzed by linear trap quadropole (LTQ) Orbitrap Velos mass spectrometer (Thermo Fisher Scientific, Waltham, MA, USA) equipped with an Advion nanomate electrospray ionization (ESI) source (Advion, Ithaca, NY, USA). Peptides (500 ng) were eluted from a C18 column (100-μm id × 2 cm, Thermo Fisher Scientific) onto an analytical column (75-μm ID × 10 cm, C18, Thermo Fisher Scientific) using a 180 min gradient with 99.9% acetonitrile, 0.1% formic acid at a flow rate of 400 nL/min and introduced into an LTQ-Orbit rap. Data dependent scanning was performed by the Xcalibur v 2.1.0 software [35] using a survey mass scan at 60,000 resolution in the Orbitrap analyzer scanning mass/charge (*m*/*z*) 400–1600, followed by collision-induced dissociation (CID) tandem mass spectrometry (MS/MS) of the 14 most intense ions in the linear ion trap analyzer. Precursor ions were selected by the monoisotopic precursor selection (MIPS) setting with selection or rejection of ions held to a ±10 ppm window. Dynamic exclusion was set to place any selected *m*/*z* on an exclusion list for 45 s after a single MS/MS. Tandem mass spectra were searched against a *Streptococcus pneumoniae* serotype4 strain ATCC BAA/TIGR4 fasta protein database downloaded from UniProtKB to which common contaminant proteins (e.g., human keratins obtained at ftp://ftp.thegpm.org/fasta/cRAP) were appended. All MS/MS spectra were searched using Thermo Proteome Discoverer 1.3 (Thermo Fisher Scientific) considering fully tryptic peptides with up to two missed cleavage sites. Variable modifications considered during the search included methionine oxidation (15.995 Da), and cysteine carbamidomethylation (57.021 Da). Peptides were identified at 99% confidence with XCorr score cutoffs [36] based on a reversed database search. The protein and peptide identification results were visualized with Scaffold v 3.6.1 (Proteome Software Inc., Portland, OR, USA). Protein identifications with a minimum of two peptides identified at 0.1% peptide false discovery rate (FDR) were deemed correct. Significant changes in protein expression between *ΔcadA* and TIGR4 were identified by Fisher’s exact test at a *p*-value of ≤0.054 and fold change of ±1.3. Fold changes in protein expression were calculated using weighted normalized spectra with 0.5 imputation value. Various bioinformatics resources such as DAVID [37], KEGG [38] and STRING [39] were utilized to determine the functions of the identified proteins. The PRoteomics IDEntifications (PRIDE) database is a centralized, standards compliant, public data repository for proteomics data. The mass spectrometry proteomics data from this study is deposited to the ProteomeXchange Consortium via the PRIDE partner repository [40] with the dataset identifier PXD008621.

### 2.5. Quantitative Real Time PCR

Gene expression in *ΔcadA* was measured by quantitative reverse transcription-PCR (qRT-PCR). The primers used for qRT-PCR are listed in Table 1. All primers were validated by performing a melt curve analysis with SYBR Green (Thermo Fisher Scientific Waltham, MA, USA) to ensure the amplification of a single specific product. In brief, total RNA was purified from mid-log phase TIGR4 and *ΔcadA* grown in THY (*n* = 3) using the RNeasy Midi kit and QIAcube (Qiagen, Valencia, CA, USA). Purified total RNA (7.5 ng/reaction) was transcribed into cDNA and qRT-PCR was performed using the SuperScript III Platinum SYBR Green One-Step qRT-PCR Kit (Thermo Fisher Scientific, Waltham, MA, USA) as previously described [34]. Relative quantification of gene expression was determined by using the Stratagene Mx3005P qPCR system (Agilent, Santa Clara, CA, USA). Expression of target genes, *speE*, *potD*, *cps4A*, *aguA*, *lys9,* and *nspC* was normalized to the expression of *gyrB* and fold change determined by the comparative C_T_ method.

## 3. Results

### 3.1. Impact of ∆cadA on Pneumococcal Growth

**Figure 1:**
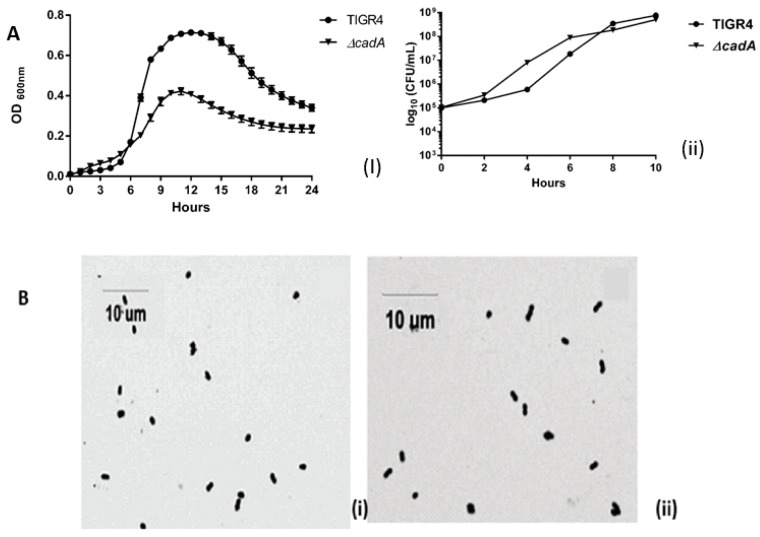
Growth of TIGR4 and *ΔcadA* and Gram stain morphology in vitro. (**A**) Growth of TIGR4 and *ΔcadA* in THY (*n* = 3) was monitored by measuring absorbance 600 nm (**i**) and viability (**ii**) was estimated by plating on blood agar plates (BAP) for colony forming units (CFU) enumeration. (**B**) Morphology of TIGR4 (**i**) and *ΔcadA* (**ii**) was observed by Gram staining.

*S. pneumoniae* TIGR4 and *ΔcadA* had comparable growth kinetics in THY, as reported previously [24]. The deletion mutant had a shorter lag phase than TIGR4 and had a lower cell density at stationary phase (Figure 1A(i)). There is evidence that pneumococcal serotypes with high colonization prevalence have a short lag phase in vitro when cultured in complete medium compared to invasive serotypes [41]. The shorter lag phase in *ΔcadA* could have implications for its invasiveness that needs confirmation in future studies. Since polyamines impact a number of cellular processes that can modulate growth, it is possible that deletion of *ΔcadA* alters some of these processes that ultimately results in reduced cell density. However, there is no significant difference in the exponential growth rate between TIGR4 (0.011 min^−1^) and *ΔcadA* (0.015 min^−1^). Both TIGR4 and *ΔcadA* had comparable CFUs during growth in THY (Figure 1A(ii)). Mid-log phase TIGR4 (Figure 1B(i)) and *ΔcadA* cells (Figure 1B(ii)) showed no difference morphology as both exhibited the characteristic diplococci morphology. These results are similar to our earlier observation that deletion of lysine decarboxylase has no qualitative difference in pneumococcal growth in vitro. However, lysine decarboxylase is indispensable for survival in murine models of colonization, pneumonia and sepsis [24].

### 3.2. Lysine Decarboxylase is Required for Capsule Production in S. pneumoniae

**Figure 2:**
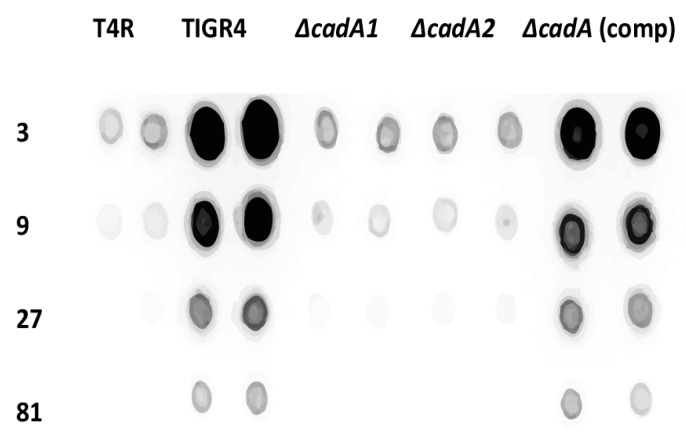
Immunoblot analysis of capsular polysaccharides in TIGR4 and mutant strains. All strains were cultured in THY supplemented with fetal bovine serum (FBS) to mid-log phase. Total capsular polysaccharide (CPS) isolated from equal number of cells for each strain, and 3× dilutions were spotted onto a nitrocellulose membrane. Membranes were probed with rabbit anti-serotype 4 sera and horseradish peroxidase (HRP)-conjugated goat anti-rabbit secondary antibody. Membranes were developed with enhanced chemiluminiscence (ECL) detection and scanned using a ChemiDoc XRS+ with Image Lab software (Bio-Rad, Hercules, CA, USA). Data from representative immunoblot from two independent colonies for each strain are shown.

Isogenic deletion of lysine decarboxylase in *S. pneumoniae* TIGR4 led to attenuation in murine models of colonization, pneumococcal pneumonia and sepsis [24]. The capsule renders pneumococci resistant to opsonophagocytosis and is essential for pneumococcal virulence [42]. Loss of the capsule associated with impaired cadaverine synthesis could explain the observed attenuation of virulence in *S. pneumoniae ∆cadA*. We compared total CPS from TIGR4, T4R, *∆cadA* and *∆cadA* complemented strains (*ΔcadA* (comp)) using serotype 4-specific CPS antibodies. Our results (Figure 2) clearly show that deletion of *cadA* results in loss of the capsule in two independently-derived mutants that lack lysine decarboxylase (*∆cadA1* [24] and *ΔcadA2* (this study)) compared to TIGR4. Both *∆cadA* deletion strains exhibit loss of the capsule (Figure 2) ruling out the possibility that the observed phenotype is due to a random change in the genome elsewhere and not specific to *cadA* deletion. This was further confirmed by complementation. CPS in *ΔcadA* is fully restored to the levels comparable to that of TIGR4 by complementation with pABG5-*cadA* construct. These results clearly demonstrate that deletion of lysine decarboxylase in *S. pneumoniae* results in the loss of CPS. Loss of the capsule could render *ΔcadA* susceptible to host defenses resulting in an attenuated phenotype in murine models of colonization and invasive disease. Impact of lysine decarboxylase on capsule synthesis in vivo needs to be evaluated in future studies.

### 3.3. Lysine Decarboxylase Effects on Pneumococcal Protein Expression

To identify pneumococcal molecular mechanisms that are responsive to lysine decarboxylase, we carried out mass spectrometry based proteomics with TIGR4 and *∆cadA*. A total of 772 proteins were identified (Supplementary Table 1) which represents 34.5% of the annotated protein coding genes in the TIGR4 genome [27]. We identified significant changes in the expression of 132 proteins of which 52 are upregulated and 80 are downregulated in *∆cadA* compared to TIGR4 (Supplementary Table 2). Molecular functions and pathways represented by the differentially expressed proteins are discussed in the following sections and shown in Table 2.

#### 3.3.1. Capsule Biosynthesis

CPS synthesis in TIGR4 is by the Wzy polymerase dependent mechanism. CPS synthesis is a multistep process that begins with the transfer of sugar-1-phosphate on the cytosolic side onto a C55 lipid undecaprenyl-phosphate (Und-P), followed by the addition of remaining sugars by glycosyl transferases to form a repeat unit. The repeat unit structure of serotype 4 CPS consists of galactose, *N*-acetylmannosamine, *N*-acetylfucosamine and *N*-acetylgalactosamine [43]. Und-P oligosaccharide repeat units are translocated to the outer face of the cytoplasmic membrane by Wzx transporter and polymerized into high molecular weight polysaccharide by Wzy polymerase. All genes involved in capsule biosynthesis are present as a single operon between *dexB* and *aliA* in pneumococcal genomes. The first four genes in the operon *cpsABCD* are important for modulation of synthesis and are common to all serotypes [44]. The rest of the genes in the operon are serotype specific. Changes in the expression of *cpsA* is a good measure of the transcription *cps* locus. We expected to see a significant reduction in *ΔcadA* in the expression of some of the enzymes that catalyze the multi-step CPS biosynthesis. Our proteomics data showed reduced expression of UDP-glucose-4-epimerase (Table 2), that catalyzes the conversion of UDP-glucose to UDP-galactose in CPS biosynthesis [45]. While the reduced expression of this protein is consistent with the observed loss of capsule, it is unlikely that this marginal change can explain the magnitude of the loss of CPS comparable to unencapsulated T4R (Figure 2) without additional changes in the pneumococcal proteome that directly or indirectly impact capsule synthesis. Expression of bifunctional protein GlmU (Table 2) was significantly downregulated. This enzyme catalyzes the last two sequential reactions in the de novo biosynthetic pathway for UDP-*N*-acetylglucosamine (UDP- GlcNAc). GlmU catalyzes the reaction that transfers an acetyl group from acetyl coenzyme A to glucosamine 6-phosphate to synthesize acetylated glucosamine 6-phosphate. NagB is an enzyme that catalyzes the conversion of glucosamine 6-phosphate to fructose 6-phosphate and is known to regulate GlmU [46]. In *∆cadA* expression of NagA and NagB, two enzymes involved in UDP-GlcNAc degradation was significantly higher compared to TIGR4. Taken together, the net effect of changes in the expression of GlmU, NagA and NagB would result in lower concentrations of UDP-GlcNAc, a precursor for UDP-ManNAc, which is a constituent of serotype 4 CPS repeat unit, and could contribute to reduced CPS synthesis in *∆cadA.*

#### 3.3.2. Polyamine Biosynthesis

**Figure 3:**
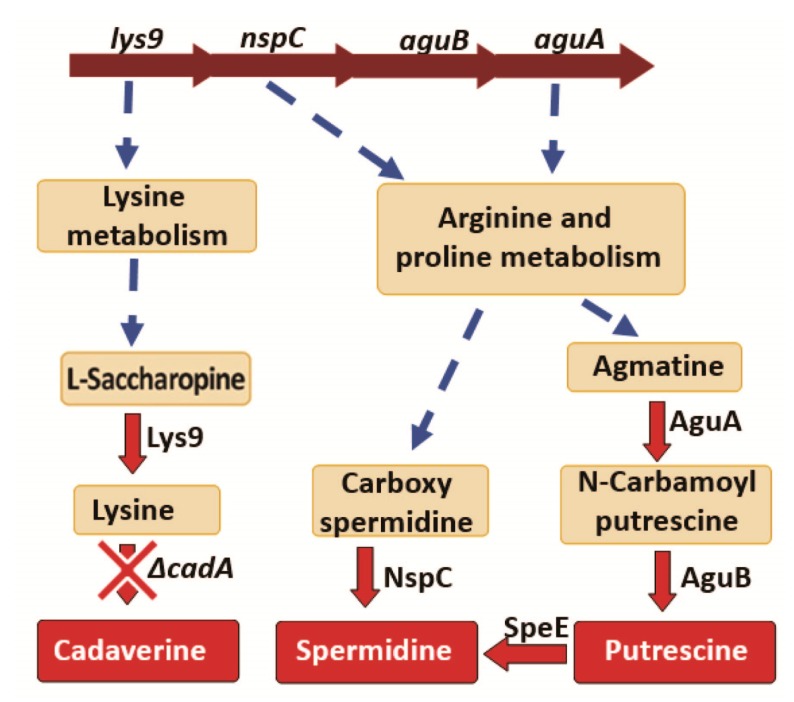
Impact of lysine decarboxylase on polyamine synthesis. Genes encoding the enzymes Lys9, NspC, AguB and AguA that catalyze reactions in the polyamine biosynthesis pathways are arranged as a single operon in the genome, and Transcription of this operon is downregulated in *ΔcadA*. Reactions that involve multiple steps are represented by a broken arrow. We identified reduced expression of *lys9*, *nspC*, *aguA* and *speE* in *ΔcadA* compared to TIGR4 by qRT-PCR. Expression of Lys9 and NspC proteins were reduced in lysine decarboxylase impaired pneumococci.

Deletion of lysine decarboxylase in *S. pneumoniae* resulted in a significant decrease in the expression of Lys9, Asd, DapA, DapB, Hom, DapH and SP_2096 involved in the biosynthesis of lysine, the substrate for *cadA* (Table 2). Expression of *N*-carbamoylputrescine amidase which catalyzes the synthesis of putrescine from *N*-carbamoylputrescine in the arginine and proline metabolism was significantly downregulated. Expression of NspC which catalyzes the synthesis of spermidine from carbamoyl spermidine was also significantly downregulated (Table 2). The net effect of these protein expression changes in polyamine biosynthesis pathways in *ΔcadA,* is expected to result in reduced intracellular concentrations of cadaverine, putrescine and spermidine (Figure 3). We reported reduced intracellular concentrations of cadaverine, putrescine and spermidine in *ΔcadA* [24] previously. Results from this study explain this observed impact on intracellular polyamine concentrations in *ΔcadA*.

#### 3.3.3. Peptidoglycan

The rigid, stable shape of bacteria is provided by the peptidoglycan layer which is made of *N*-acetylmuramic acid-(β-1, 4)-*N*-acetylglucosamine (MurNAc-GlcNAc) disaccharides cross-linked by peptides. The peptidoglycan layer, teichoic acid (TA) and lipoteichoic acid (LTA) constitute the cell wall of Gram-positive bacteria such as *S. pneumoniae* [47]. Both LTA and TA contain phosphorylcholine (PC), which plays a major role in *S. pneumoniae* adhesion and also forms attachment of choline binding surface proteins (CBPs) [48]. The peptidoglycan layer provides attachment for many structural components including the polysaccharide capsule [49] and it is important in the adhesion of *S. pneumoniae* to the host tissues. Our results show reduced expression of penicillin-binding protein 2X (Table 2), which is involved in peptidoglycan biosynthesis that contributes to bacterial cell division and growth [50,51]. Choline kinase, an enzyme which catalyzes the synthesis of PC from choline was downregulated in *ΔcadA* relative to TIGR4. In some Gram-positive pathogens, choline kinase is known to be important for the production of cell wall elements and LTA [52]. PC is necessary for the adherence of *S. pneumoniae* during the transition from colonization to invasive disease [48]. Reduced choline kinase expression in *ΔcadA* could also modulate virulence.

#### 3.3.4. ABC Transporters

Our results show that expression of ABC transporters that bind metal ions is significantly altered in *∆cadA.* Manganese, a transition metal ion is a prosthetic group in superoxide dismutase, has direct antioxidant properties and is known to be important for pneumococcal physiology. An ABC-type permease PsaBCA [53] transports manganese and *psa* mutants are avirulent [54] due to their hypersensitivity to oxidative stress [51]. Expression of Manganese ABC transporter-substrate-binding lipoprotein (PsaA) and Manganese ABC transporter, ATP -binding protein (PsaB) is significantly higher in *∆cadA* (Table 2). An ABC transporter potentially responsible for iron-siderophore transport (FhuD), specifically ferric hydroxamate is significantly lower in *∆cadA* compared to TIGR4. Iron is a critical cofactor for many enzymes, and uptake and efflux of this critical micronutrient from different host niches that differ in the quantity and form of iron is an important aspect of pneumococcal pathogenesis. Reduced expression of FhuD could impair iron homeostasis and have an impact on virulence. An alternate explanation involves *∆cadA* response to oxidative stress by reducing uptake of iron due to reduced expression of FhuD. High intracellular iron concentrations can result in an increase in oxidative stress [55] and *ΔcadA* with reduced intracellular polyamine concentrations would be more susceptible to oxidative stress [21]. Increased expression of PsaA and PsaB could also be in response to increased susceptibility to oxidative stress in *∆cadA*.

Expression of five ABC transporters, four of which are involved in oligopeptide trafficking (SP_2073, OppA, OppD, OppB and OppF) were downregulated in *ΔcadA* (Table 2). Reduced expression of oligopeptide transporter proteins could significantly alter transport of substrates that would in turn affect biosynthesis of amino acids, polyamines and other cellular components. There was reduced expression of lysine-transfer RNA (tRNA) ligase, a protein involved in amino acid metabolism. Expression of RplU, which binds to 23S ribosomal RNA (rRNA) in the initial stages of protein translation and ribosome maturation factor (RimP), which modulates final stages of translation was significantly reduced in *ΔcadA*. Our results show that lysine decarboxylase deficiency results in reduced expression of a number of proteins involved in amino acid transport and metabolism which could impair pneumococcal growth and fitness. For survival in the host, pathogenic bacteria have to cope with phosphate (Pi) limiting or enriched host microenvironments. Phosphate is the component of nucleic acids, phospholipids and energy storage (ATP). Bacteria acquire phosphate by Pi-specific transport systems. Under Pi limitation Pho regulon is activated and results in Pi import through an ABC transporter complex PstSACB [56]. Our data showed increased expression of PstS2, PstB3, PstC and PhoU (Table 2) which can impact Pi homeostasis. Low Pi is known to alter virulence factor expression in a number of pathogenic bacteria including *E. coli* [57]. It is known that activation of Pho regulon in pathogenic bacteria under Pi starvation conditions, activates oxidative stress response through mechanisms that are yet to be described [56]. Increased expression of Pst system could be part of oxidative stress response and possibly contribute to altered virulence in *ΔcadA* through mechanism that are not known at present.

#### 3.3.5. Pentose Phosphate Pathway

The ability of bacterial pathogens to survive in the host largely depends on acquiring nutrients and adapting their metabolism to different host microenvironments. Pneumococci have the ability to utilize a variety of carbohydrates as carbon sources via Embden-Meyerhof-Parnas (EMP) pathway (glycolysis) and pentose phosphate pathway (PPP) [27]. Pyruvate and ATP are the end products of glycolysis. The oxidative branch of PPP generates ribulose 5-phosphate and reduced nicotinamide adenine dinucleotide phosphate (NADPH) while the non-oxidative branch generates a number of sugar phosphates that provide precursors for nucleotide, amino acid and vitamin B6 synthesis. Our data supports increased carbon flux through the non-oxidative branch of PPP due to the observed increase in the expression of transketolase (Tkt), an enzyme that catalyzes the interconversion of sugar-phosphates in the pathway. Expression of all proteins SgaR2, SgaB2, SgaT2, including TktN and TktC, encoded in a single regulon belonging to BgIG family transcriptional regulator is higher in *∆cadA* (Table 2). We also identified a significantly higher expression for one of the two general proteins of PTS (phosphotransferase system), phosphocarrier protein HPr (PtsH) which could support the proper functioning of PTS transport systems in the BgIG regulon in *∆cadA*. A shift in metabolism towards PPP is often in response to oxidative stress to maintain NADH/NADPH redox homeostasis and to synthesize ribose-5-phosphate for DNA repair [58].

#### 3.3.6. Carbohydrate Metabolism

Our results show increased expression of proteins that are involved in galactose and tagatose catabolism. Expression of LacB is higher in *ΔcadA* (Table 2) which generates the substrate tagatose 6-phosphate for the enzyme LacD. Enzymatic action of LacB and LacD would result in higher levels of glyceraldehyde 3-phosphate, which can be channeled into PPP by transaldolase and transketolase. We identified reduced expression of catabolite control protein A (CcpA), a protein that regulates carbon catabolite repression in our lysine decarboxylase mutant. CcpA is known to control the expression of a number of virulence factors in Gram-positive bacteria [59]. For instance, in *S. pneumoniae,* CcpA contributes to sugar metabolism and virulence [60].

### 3.4. Measurement of Gene Expression in ΔcadA

#### 3.4.1. Capsule Biosynthesis

We did not identify major differences in the expression of proteins that catalyze different steps in CPS synthesis. To determine whether CPS synthesis is regulated at the transcriptional level, we compared *cps4A* mRNA expression between TIGR4 and *ΔcadA* by qRT-PCR. Our results show a significant reduction in the expression of *cps4A* (Table 3), which could explain reduced CPS synthesis in *ΔcadA*.

#### 3.4.2. Polyamine Synthesis and Transport

Our proteomics data identified significantly reduced expression of proteins involved in the biosynthesis of putrescine, spermidine and cadaverine (Figure 2). The genes encoding proteins NspC, Lys9, AguA and AguB are predicted to constitute a single operon in the genome [61]. Based on our proteomics results, we expected to see reduced expression of polyamine biosynthesis (*lys9*, *aguA*, and *nspC*) genes and our qRT-PCR data shows reduced expression of these genes in *ΔcadA* (Table 3). Impaired lysine decarboxylase also resulted in a significant reduction in the expression of spermidine synthase (Table 3). Pneumococci can compensate for reduced polyamine synthesis by increasing the import of extracellular polyamines. Extracellular polyamine uptake in pneumococci is predicted to be via a single ABC transporter, organized as a four gene operon, *potABCD* [62]. The proposed structure of the putrescine/spermidine transporter has PotD, an extracellular substrate binding domain that binds polyamines, PotB, and PotC which form transmembrane channels that transport polyamines and PotA that is a membrane associated cytosolic ATPase [23]. We did not detect polyamine transport proteins in our proteomics data. We measured the expression of *potD* mRNA in *ΔcadA* and observed a significant decrease (Table 3), which would further contribute to reduced intracellular concentrations of putrescine and spermidine, putative substrates for the PotABCD transporter.

## 4. Discussion

Polyamines are important for host-pathogen interactions during bacterial infections. Polyamines in pathogenic bacteria play an important role in physiological stress responses and adaptation to growth in vivo. Putrescine is a constituent of the cell wall in a number of Gram-negative bacteria, such as *Salmonella enterica*, *E. coli* and *Proteus mirabilis* [63,64], while cadaverine is covalently linked to the peptidoglycan layer of *Veillonella alcalescens* [65]. Spermidine modulates autolysis and ion trafficking across the cell membrane in *S. pneumoniae*, protecting pneumococci from cationic antimicrobial compounds [7]. Cadaverine regulates porins that control the permeability of membranes [30], and enables *E. coli* to survive acidic stress [66]. Current literature clearly demonstrates that deletion of polyamine synthesis and/or transport in pathogenic bacteria (including pneumococci) leads to reduced virulence in animal models [24,67,68]. To date, studies that describe specific host innate immune mechanisms induced by pathogenic bacteria with altered polyamine metabolism, or specific effects of impaired polyamine metabolism on pathogen molecular mechanisms are largely unknown. A few examples of specific roles of polyamines in bacterial pathogens include the following: in intracellular pathogen *Shigella*, cadaverine is shown to be important for reducing enterotoxic activity [69], inhibiting trans-epithelial migration of polymorphonuclear neutrophils, increasing survival in macrophages, and enhancing antioxidant defenses in vitro [20]. *Salmonella typhimurium* mutant deficient in polyamine biosynthesis has reduced invasive potential and survival in epithelial cells in vitro and is attenuated in a mouse model of typhoid fever [70]. In *Yersinia pestis*, loss of intracellular spermidine and putrescine affects biofilm formation and biosynthesis defective *Y. pestis* is less virulent in a murine model of bubonic plague [71]. When *Francisella tularensis* is cultured in the presence of spermine or spermidine prior to macrophage infection assays, there is reduced pro-inflammatory response in vitro [55]. Thus altered polyamine metabolism has varying impacts on bacterial virulence.

Invasive infections caused by *S. pneumoniae*, a commensal in the nasopharynx, pose significant risk to human health. The available polysaccharide-based conjugate vaccines are effective in reducing vaccine serotypes in population, but ultimately lead to serotype replacement [72] from a reservoir of more than 90 capsular serotypes [3]. The critical role of the upper respiratory microbiota and the consequence of its perturbation in various pathophysiological conditions mandates a cautious approach to drug and vaccine discovery. Anti-virulence strategies that target genes/proteins that are necessary for fitness during invasive infection can offer serotype-independent coverage without impacting nasopharyngeal colonization, that is, disarm, but not eradicate, pneumococci [68]. Specific aspects of bacterial physiology and metabolism that allow pathogens to adapt to the host are a promising avenue for the discovery of novel therapeutics. Intracellular polyamine concentrations are tightly regulated by biosynthesis, transport and degradation. Polyamine transport and synthesis genes are conserved in pneumococci. Deletion of the polyamine transport *(potABCD)* operon or spermidine synthase and lysine decarboxylase biosynthesis genes had no significant impact on pneumococcal growth in vitro [24]. However, enhanced bacterial clearance in murine models of colonization, pneumococcal pneumonia, and sepsis were seen with mutant strains [24]. In a murine model of pneumococcal pneumonia, *∆potABCD* failed to elicit host defenses that are intact in TIGR4, and was cleared more efficiently by opsonophagocytosis by neutrophils [34]. TIGR4 cultured in vitro (in THY) had spermidine as the most abundant intracellular polyamine, followed by cadaverine and putrescine [24]. Polyamine transport and metabolism impaired *∆potABCD*, *∆cadA* and *∆speE* showed reduced levels of spermidine, cadaverine and putrescine, relative to TIGR4. [24].

**Figure 4:**
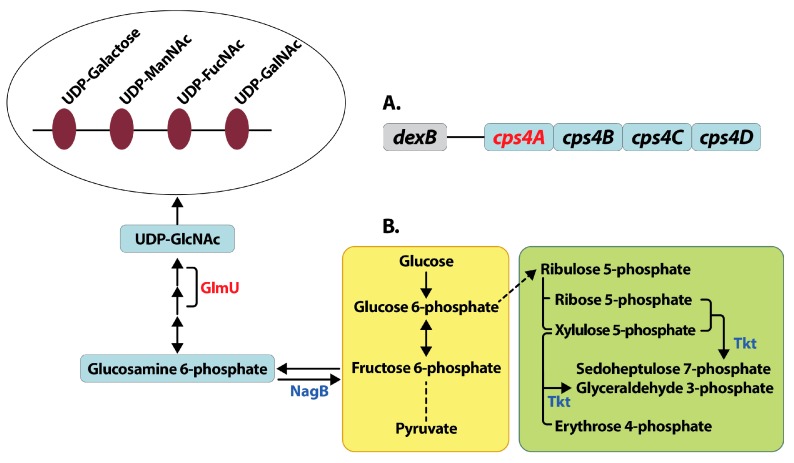
Mechanisms for reduced capsule synthesis in lysine decarboxylase deficient pneumococci. Deletion of lysine decarboxylase in pneumococci results in reduced capsule compared to wild type TIGR4 strain. Reduced capsule synthesis could be due to the reduced expression (shown in red) of capsular polysaccharide biosynthesis gene *cps4A*, the first gene in the *cps* locus in *S. pneumoniae* TIGR4 (**A**). The first four genes from the *cps* locus adjacent to the gene *dexB* that are conserved in all pneumococcal serotypes are shown. Our proteomics data indicates a shift in central metabolism (**B**) from glycolysis (yellow box) to the non-oxidative branch of pentose phosphate pathway (green box), due to increased expression (shown in blue) of transketolase (Tkt,). Reactions that involve multiple steps are represented by a broken line. Reduced expression of GlmU involved in the synthesis and increased expression of NagB involved in the degradation of UDP-GlcNAc could result in reduced concentration of UDP-GlcNAc. UDP-GlcNAc is a precursor for UDP-ManNAc, an acetylated sugar that is the constituent of the 4-sugar repeat unit of capsular polysaccharide in capsular serotype 4 (open oval). The net effect of these changes in pneumococcal gene and protein expression could result in the observed reduction in capsule biosynthesis in *ΔcadA*.

Here we report in vitro characterization of lysine decarboxylase deficient pneumococci. Our results clearly demonstrate loss of CPS in *∆cadA*. This loss of the capsule is specific to the deletion of the *cadA* gene, as complementation of the wild type gene restored capsule, comparable to that of TIGR4. Invasive pneumococcal serotypes can resist complement mediated opsonophagocytosis by neutrophils due to the presence of the capsule [71,73]. All three complement pathways are activated for opsonophagocytosis [73,74]; classical, lectin and alternative. Activation of the classical pathway is by antibody (including non-specific immunoglobulin M (IgM) produced during infection), or by C-reactive protein (CRP), an acute phase protein. Steric inhibition of the interaction between complement components and the Fc portion of immunoglobulins by the capsule enables pneumococci to evade this host defense. The alternative pathway is constitutively activated at low levels by complement protein C3b, and capsule-mediated resistance to opsonophagocytosis also includes decreased cleavage of C3b to iC3b [75]. Pneumococci regulate capsule expression as they invade host tissues. In the initial stages of colonization of the nasopharynx, they express the capsule to evade mucus, and in the subsequent stage of adhesion, the capsule is downregulated to expose surface adhesins for adherence and colonization. The invasive phase requires upregulation of the capsule to resist opsonophagocytosis [76]. Some of the known regulatory mechanisms for CPS synthesis involve phase variation [77], deletion of *pgdA* and *adr*, acetylases of peptidoglycan and increased transcription of *cpsA* [78] and deletion of pyruvate oxidase (*spxB*) that all lead to increased CPS [79], mutations in *arcD* that result in reduced CPS [80], and tyrosine phosphorylation of CpsBCD proteins that modulate CPS levels [81]. Here, for the first time, we show that polyamines, specifically cadaverine modulate CPS synthesis. Inactivation of lysine decarboxylase in pneumococci results in reduced capsule synthesis. The mechanisms that result in reduced CPS synthesis are due to the combinatorial effects on the regulation of UDP-GlcNAc synthesis and degradation (Figure 4) that could reduce the availability of UDP-ManNAc of the CPS repeat unit in serotype 4 pneumococci with concomitant transcriptional downregulation of *cps4A* gene. A moderate reduction in *cpsA* expression in TIGR4 capsule promoter mutant resulted in twofold reduction in CPS [78]. In *∆cadA*, there is a twofold reduction in the expression of *cps4A mRNA* (Table 3), which would be expected to have a greater impact on CPS synthesis. The intersection between central metabolism and virulence is becoming evident for many bacterial pathogens including pneumococci. As pneumococci sense and adapt to different host niches, they modulate capsule synthesis which requires a shift in the metabolism towards increased synthesis of precursors for CPS synthesis. A recent study showed that reduced acetyl-coA levels result in loss of capsule [56], supporting the link between central metabolism and capsule formation.

Given the well-established role of polyamines in bacterial adaptation to oxidative stress [24], the reduced intracellular polyamines due to reduced expression of enzymes catalyzing synthesis (proteomics data, Table 2) and transport (qRT-PCR data, Table 3) could explain the observed shift in metabolism that increases carbon flux through the pentose phosphate pathway. Reduced uptake of iron and upregulation of manganese transporter proteins, known pneumococcal virulence factors linked to oxidative stress response support this idea. It is possible that increased oxidative stress in *∆cadA* results in metabolic adaptation that diverts the energy from capsule production towards stress responses. With data presented here, it is difficult to distinguish between correlation and causation due to impaired cadaverine synthesis. Future studies that utilize additional omics approaches and data analysis in an integrated manner are required to deconvolute the complexity of pneumococcal response to altered polyamine metabolism. Although we have begun to identify some of the molecular mechanisms that are responsive to polyamines in pneumococci that govern virulence, annotation of polyamine metabolism genes is limited in pneumococci. Our knowledge of polyamine metabolism in pathogenic bacteria and specific effects of polyamines on bacterial translation relies heavily on studies from *E. coli*. Basic biochemistry pertaining to substrate specificity of the transporters and biosynthesis genes is yet to be determined. Our data shows that reduced expression of putrescine and spermidine biosynthesis in *ΔcadA* could ultimately result in the altered intracellular polyamines that we reported earlier [24]. Future studies focused on determining individual contribution of these polyamine biosynthesis genes to pneumococcal virulence are necessary for a comprehensive description of the role of polyamine synthesis in pneumococcal pathogenesis. Specific effects of polyamines on protein synthesis in bacteria are known. Studies in *E. coli* have identified a “polyamine modulon”, a set of 17 genes whose translation is enhanced by polyamines [82]. Mechanisms by which polyamines increase protein synthesis include formation of the initiation complex due to structural changes in the Shine-Dalgarno sequence and the initiation codon AUG, initiation from inefficient initiation codon, and +1 frame shifting and suppression of nonsense codons. Here we report altered expression of a number of pneumococcal proteins in response to impaired lysine decarboxylase. It is possible that some of these proteins are regulated by polyamines utilizing the mechanisms described in *E. coli*. Future studies focused on understanding the specific mechanisms utilized by polyamines to impact protein expression can result in mechanistic insights into the impact of altered metabolism on pneumococcal virulence. Despite these knowledge gaps, our previous work has clearly established the importance of polyamine transport and synthesis genes in pneumococcal pathogenesis. In this study we show that impaired polyamine synthesis results in reduced capsule synthesis. This foundational knowledge at the intersection of polyamine metabolism and pneumococcal virulence reinforces the need for future mechanistic studies focused on developing novel therapeutics targeting polyamine metabolism in pneumococci.

## Figures and Tables

**Table 1:** Sequences of primers used in this study.

Primer	Sequence * (5′→3′)	Experiment
*cadAF1*	AGCAAATATAAACCCGAGTAAAAA	Mutagenesis
*cadAR1*	CAGGTACCGCTTGTGACCTGGAACATC	Mutagenesis
*cadAF2*	CAGAGCTCGTTTCGGTTTGCGATTTT	Mutagenesis
*cadAR2*	GATCTTCCGTCCCTTGGAG	Mutagenesis
*cadAF-XbaI*	TTCCCCGGGCCGTGCGAAAATCATCGCC	Complementation
*cadAR-SacI*	ATTCGAGGAAGACAGAGGTGTACTATTC	Complementation
*gyrBF*	CCGTCCTGCTGTTGAGACC	qRT-PCR
*gyrBR*	GTGAAGACCACCTGAAACCTTG	qRT-PCR
*potDF*	AAACCTGAAAATGCTCTCCAAAATG	qRT-PCR
*potDR*	CCTTATCTTCCTTTGTTTCCTCTGG	qRT-PCR
*cps4AF*	TCAAGTCAAGTCAGAATACCGATTTG	qRT-PCR
*cps4AR*	TCAAAGACACTATTTAGGACAATGGC	qRT-PCR
*speEF*	TGCGGATGATTTCGTCTACAATG	qRT-PCR
*speER*	CCAGTTCAGGATAGAGGGTTAATAC	qRT-PCR
*aguAF*	GCTTAGTCCTGGTCGCAATC	qRT-PCR
*aguAR*	CTGGGGATCATTTTCGTCAT	qRT-PCR
*lys9F*	GGCTTGACTGCTCTTCTTGG	qRT-PCR
*lys9R*	AGTAAGAACCTGGCGCAGAA	qRT-PCR
*nspCF*	ATGTATTTGCGCCTGCTTTC	qRT-PCR
*nspCR*	TGGTGCACAAGGGTCATAGA	qRT-PCR

* underlined sequence complementary to *Streptococcus pneumoniae* TIGR4 chromosomal DNA. qRT-PCR: quantitative reverse transcription-PCR.

**Table 2:** Significant changes in *ΔcadA* proteome compared to TIGR4.

Description	Protein	*ΔcadA*/TIGR4 (Fold Change)	Function
*N*-carbamoylputrescine amidase	*SP_0922	−10.0	Putrescine biosynthesis
Carboxynorspermidine decarboxylase	NspC	−5.0	Spermidine biosynthesis
Homoserine dehydrogenase	Hom	−1.4	Lysine biosynthesis
4-hydroxy-tetrahydrodipicolinate synthase	DapA	−10.0	Lysine biosynthesis
4-hydroxy-tetrahydrodipicolinate reductase	DapB	−2.5	Lysine biosynthesis
*N*-acetyldiaminopimelate deacetylase	SP_2096	−2.5	Lysine biosynthesis
Saccharopine dehydrogenase	Lys9	−25.0	Lysine biosynthesis
Aspartate-semialdehyde dehydrogenase	Asd	−25.0	Lysine biosynthesis
2,3,4,5-tetrahydropyridine-2-carboxylate *N*-Succinyl transferase	DapH	−1.7	Lysine biosynthesis
50S ribosomal protein L21	RplU	−5.0	Regulation of protein elongation
Ribosome maturation factor	RimP	-5.0	Regulation of protein maturation
Lysine-tRNA ligase	LysS	−3.3	Amino acid metabolism
Iron-compound ABC Transporter	FhuD	−50.0	Iron complex ABC transporter
Phosphate-binding protein PstS 2	PstS 2	41.0	Phosphate ion transport
Phosphate import ATP-binding protein PstB 3	PstB 3	36.0	Phosphate ion transport
Phosphate transport system permease protein	PstC	7.0	Phosphate ion transport
Phosphate-specific transport system accessory protein PhoU homolog	PhoU	43.0	Phosphate ion transport
ABC transporter, ATP-binding/permease protein	SP_2073	−3.3	Oligopeptide ABC transporter
Oligopeptide binding protein	OppA	−25.0	Oligopeptide ABC transporter
Oligopeptide transport ATP-binding protein	OppD	−1.4	Oligopeptide ABC transporter
Oligopeptide transport ATP-binding protein	OppF	−1.7	Oligopeptide ABC transporter
Oligopeptide transport system permease protein	OppB	−1.7	Oligopeptide ABC transporter
Manganese ABC transporter-substrate-binding lipoprotein	PsaA	2.4	Oxidative stress
Manganese ABC transporter, ATP -binding protein	PsaB	6.8	Oxidative stress
Penicillin-binding protein 2x	Pbp2X	−2.5	Peptidoglycan biosynthesis
Choline kinase	Pck	−2.0	Cell wall biosynthesis
UDP-glucose 4-epimerase	GalE-1	−1.3	Carbohydrate metabolism
Tagatose 1,6-diphosphate aldolase	LacD	1.4	Carbohydrate metabolism
Galactose-6-phosphate isomerase subunit	LacB	2.1	Carbohydrate metabolism
Catabolite control protein A	CcpA	−2.5	Carbohydrate metabolism
Bifunctional protein	GlmU	−1.7	UDP- GlcNAc synthesis
*N*-acetylglucosamine-6-phosphate deacetylase	NagA	1.4	*N*-acetylglucosamine degradation
*N*-acetylglucosamine-6-phosphate deaminase	NagB	2.1	*N*-acetylglucosamine degradation
Transketolase, C-terminal subunit	TktC	67.0	Pentose phosphate pathway
Transketolase, N-terminal subunit	TktN	46.0	Pentose phosphate pathway
Ascorbate-specific PTS, EIIC component	SgaT2	31.0	Ascorbate utilization
Ascorbate-specific PTS system, EIIB component	SgaB2	32.0	Ascorbate utilization
Phosphocarrier protein HPr	PtsH	21.0	Phosphotransferase system (PTS)

*: locus tag ID; ABC: ATP binding cassette; ATP: Adenosine triphosphate; UDP: uridine diphosphate; GlcNac: N-acetylglucosamine; PTS: phosphotransferase system.

**Table 3:** Changes in gene expression in *ΔcadA* compared to TIGR4.

Gene	Description	*ΔcadA*/TIGR4 (Fold change)	*p*-Value
*potD*	Spermidine/putrescine ABC transporter, spermidine/putrescine-binding protein	−2.0	1.93E−04
*speE*	Spermidine synthase	−27.0	1.29E−06
*cps4A*	Capsular polysaccharide biosynthesis protein 4A	−2.0	2.03E−07
*lys9*	Saccharopine dehydrogenase	−26.0	3.83E−12
*nspC*	Carboxynorspermidine decarboxylase	−34.0	4.70E−10
*aguA*	Agmatine deiminase	−30.0	2.87E−12

## Supplementary Materials

The following are available online at www.mdpi.com/2076-3271/6/1/8/s1. Table S1: Proteins identified by mass spectrometry, Table S2: Significant changes in *∆cadA* protein expression compared to TIGR4.
